# Impact of course length on swimming performance across age groups and swimming strokes

**DOI:** 10.3389/fspor.2025.1631870

**Published:** 2025-09-15

**Authors:** Javier Iglesias García, Francisco Hermosilla-Perona, Tomohiro Gonjo, Daniel Juárez Santos-García

**Affiliations:** ^1^ AExPH, facultad de ciencias biomédicas y de la salud, Universidad Alfonso X el Sabio Villanueva de la Cañada, Madrid, Spain; ^2^ Sport Training Laboratory, University of Castilla-La Mancha, Toledo, Spain; ^3^ Facultad de Ciencias de la Vida y la Naturaleza, Universidad Nebrija, Madrid, Spain; ^4^ Institute of Life and Earth Sciences, School of Energy, Geoscience, Infrastructure and Society, Heriot-Watt University, Edinburgh, United Kingdom

**Keywords:** performance analysis, swimming, short course, long course, stroke

## Abstract

**Introduction:**

Swimmers typically achieve faster times in the same distance events in short course (SC) than in long course (LC) due to the higher number of turns in SC; however, the influence of age and sex on performance differences between SC and LC events remains unclear.

**Methods:**

This study aimed to examine the differences in the top 200 seasonal times between SC and LC in the 50, 100 and 200 m backstroke, breaststroke, butterfly, freestyle and individual medley events (200 and 400 m). Top 200 Spanish seasonal times of four age groups were considered for both sexes between two seasons. A three-way ANOVA with *post-hoc* analysis was performed to assess the impact of age, sex, and event type on the time differences between SC and LC.

**Results:**

The results indicated that older swimmers showed greater differences between SC and LC times compared to other age groups in males and females (*p* < 0.05). In backstroke and breaststroke events, similar patterns were observed with higher differences between SC and LC compared to butterfly and freestyle, especially in 100 m and 200 m events (*p* < 0.05).

**Discussion:**

These differences should be taken into consideration by coaches and swimmers when establishing differences in performance depending on the pool length.

## 1 Introduction

Swimming competitions regulated by the International Swimming Federation (World Aquatics) take place in both short-course (SC; 25 m) and long-course (LC; 50 m) pools. Upon examining the world records established in SC and LC, it is evident that swimmers consistently achieve faster times in SC. Throughout the season, swimmers typically compete in short course events held mainly between September and March (autumn and winter), and in long course events, which usually take place from March to August (spring and summer) (1).These differences are attributed to the increased number of turns performed for any given swimming distance (2), leading to an extended underwater swimming distance where swimmers travel at a higher velocity than in the free-swimming phase (2, 3).

Swimming turns are considered a key element in overall race performance, both in short and long distances (3, 4). These turns are classified into tumble turns and open turns. Tumble turns are utilized in freestyle and backstroke competitions and entail a forward roll towards the wall, with both feet pushing off from it. Open turns are employed in breaststroke and butterfly events, involving a touch of the wall with both hands, body rotation, and subsequent propulsion with the feet (5), In individual medley, it has been shown that turns in butterfly and backstroke are the fastest, while breaststroke, being the slowest, offer the greatest potential for performance improvement (6). However, the influence of turns on the overall performance depends on the strokes and sexes. For example, males perform a longer underwater swimming distance than females in 100 m and 200 m events across all strokes (7). In addition, the underwater swimming distance for freestyle evens is shorter compared to butterfly, backstroke, and breaststroke, which means that the turn performance has a greater contribution to the final performance in these three strokes compared with freestyle (7).

Turn performance has a significant impact on overall performance, with the speed gains from wall push-offs and undulatory swimming playing a crucial role in the performance differences observed between swims performed in pools of varying lengths (8, 9). Additionally, these turns result in physiological and biomechanical differences between SC and LC events, such as shorter stroke length observed in SC and higher heart rate and lactate concentrations recorded in LC (2, 10–13). Also, the age of swimmers is a factor to consider, as adult swimmers produce 13.8% more blood lactate in LC pools compared to SC pools when swimming at the same intensity and distance than young swimmers (11). Performance differences between SC and LC swimming may be influenced by the maturational level of the swimmers. In prepubertal stages, training programs typically focus on developing aerobic capacity, which may contribute to more uniform performance between SC and LC. However, further research is needed to better understand how these characteristics influence the differences observed between the two conditions (14). As maturation progresses, the development of the anaerobic system increases lactate production, potentially accentuating these differences. Additionally, anthropometric factors such as height, fat-free mass, and arm span evolve with age and may affect swimming biomechanics and efficiency (15, 16).

A previous study has analyzed the performance differences between SC and LC in freestyle events between age-group and senior swimmers. The study suggests that the differences between SC and LC swimming are not consistent throughout a swimmer's career. Therefore, when evaluating swimmers' performance using SC results, they should be adjusted based on the swimmers’ age (17). However, the impact of SC vs. LC on performance remains unclear for the other swimming strokes (butterfly, backstroke, breaststroke, and individual medley events). Therefore, the aim of this study was to examine the differences in performance between SC and LC in these strokes across different age groups and male and female swimmers.

Assuming that senior swimmers might derive greater benefits from turns (e.g., improved turn techniques and efficient undulatory swimming) compared to swimmers in age groups, we hypothesized that senior swimmers would exhibit a greater time difference between SC and LC than younger swimmers. Similarly, it was also hypothesized that female swimmers would present lower differences between SC and LC than males. In addition, we hypothesized that within the age-group category, older swimmers would show greater SC-LC differences than younger swimmers, due to more advanced technical and maturational development.

## 2 Materials and methods

The analysis incorporated the best seasonal best (SB) achieved in SC and LC pools for backstroke, breaststroke, butterfly, and medley swimming events by the top 200 Spanish seasonal times during the 2017–2018 and 2018–2019 seasons. Each season spanned from September to July, and all data were extracted from the ranking of the Spanish Swimming Federation (RFEN).

Swimmers were divided into four age groups for each sex. Men were separated into Male Age Group 1 (AG1): 13–14 years; Male Age Group 2 (AG2): 15–16 years; Male Age Group 3 (AG3): 17–18 years; Male Age Group 4 (AG4): 19 years and older. Similarly, women were grouped into Female AG1: 12–13 years; Female AG2: 14–15 years; Female AG3: 16–17 years; Female AG4: 18 years and older. These groupings were based on the classification established by the Spanish and International Swimming Federation.

The World Aquatics (WA) points were used to assess the sample level, which also served to distinguish competition levels according to a recent study (18). The characteristics of all analyzed groups in the present study are detailed in Table 1.

**Table 1:** Level of the swimmers analyzed.

Age group	*N*	Males				Level	*N*	Females			Level
		Event	Time min	Time max	Fina points			Time min	Time max	Fina points	
AG-1	229	50BACK	00:30,31	00:34,63	478–320	4–5	232	00:31,38	00:39,12	635–323	4–5
	271	100BACK	01:01,59	01:10,31	588–395	4–5	266	01:06,76	01:19,15	423–380	5
	241	200BACK	02:11,74	02:33,73	613–385	5	250	02:24,22	02:50,80	625–374	4–5
	209	50BREAST	00:31,20	00:38,16	575- 314	4–5	214	00:34,73	00:44,11	600–288	4–5
	267	100BREAST	01:07,51	01:20,11	598–357	4–5	267	01:14,89	01:30,86	627–351	4–5
	225	200BREAST	02:28,18	02:56,98	614–360	4–5	231	02:39,71	03:16,87	658–341	3–4–5
	239	50FLY	00:27,33	00:34,43	541–270	4–5	242	00:30,60	00:36,66	508–295	4–5
	282	100FLY	00:58,13	01:15,76	615–278	4–5	287	01:05,59	01:19,38	605–341	4–5
	210	200FLY	02:09,04	02:57,10	625–241	4–5	205	02:22,85	03:18,24	620–231	4–5
	245	200IM	02:13,26	02:32,35	626–418	4–5	258	02:25,22	02:49,96	655–408	3–4
	198	400IM	04:47,26	05:29,61	611–404	4–5	224	05:10,13	06:14,52	633–357	4–5
AG-2	263	50BACK	00:27,54	00:31,35	638–432	4–5	277	00:29,71	00:35,09	748–448	3–4–5
	269	100BACK	00:57,18	01:04,59	734–510	3–4	291	01:02,76	01:13,79	767–468	3–4
	265	200BACK	02:05,62	02:43,72	707–319	3–4–5	278	02:14,31	02:40,24	774–453	3–4
	270	50BREAST	00:30,15	00:34,53	637–424	4–5	279	00:33,36	00:39,52	677–401	3–4–5
	275	100BREAST	01:05,12	01:14,38	666–447	4–5	290	01:10,92	01:25,45	793–422	3–4–5
	263	200BREAST	02:18,86	02:21,03	737–704	3–3	280	02:35,22	03:05,30	717–409	3–4–5
	243	50FLY	00:26,15	00:29,15	617–445	4–5	291	00:28,52	00:32,63	628–419	4–5
	274	100FLY	00:55,79	01:02,39	696–497	3–4	290	01:01,07	01:12,56	749–447	3–4–5
	249	200FLY	02:03,08	02:24,02	720–449	3–4–5	271	02:11,78	02:55,12	798–336	3–4–5
	247	200IM	02:08,00	02:21,01	706–528	3–4	267	02:18,07	02:12,69	762–492	3–4
	234	400IM	04:36,00	05:16,50	611–449	4–5	239	04:53,30	05:45,89	748–454	3–4
AG-3	289	50BACK	00:25,30	00:31,35	823–423	2–3–4–5	299	00:29,40	00:34,77	780–461	3–4
	282	100BACK	00:54,27	01:06,76	859–461	2–3–4	290	01:02,59	01:14,15	773–462	3–4
	252	200BACK	01:56,69	02:30,44	882–411	1–2–3–4	265	02:09,95	02:46,28	855–406	2–3–4
	287	50BREAST	00:29,26	00:35,11	697–403	3–4–5	304	00:32,08	00:40,25	761–380	3–4–5
	289	100BREAST	01:03,72	01:06,76	711–618	3–4	295	01:09,52	01:27,07	784–399	3–4–5
	262	200BREAST	02:16,15	02:42,48	782–460	3–4	270	02:29,25	03:15,02	806–350	2–3–4–5
	276	50FLY	00:24,59	00:28,47	742–478	3–4	299	00:27,91	00:32,03	670–443	3–4–5
	283	100FLY	00:54,03	01:03,39	766–474	3–4	303	01:00,72	01:12,79	762–442	3–4–5
	260	200FLY	02:00,10	02:43,30	775–308	3–4–5	221	02:12,76	03:11,21	772–258	3–4–5
	257	200IM	01:59,91	02:25,52	859–480	2–3–4	267	02:16,48	02:41,85	789–473	3–4
	240	400IM	04:23,09	05:29,72	796–397	3–4–5	227	04:45,35	06:01,18	813–398	2–3–4–5
AG-4	264	50BACK	00:25,34	00:30,12	819–477	2–3–4	274	00:28,46	00:35,00	851–451	2–3–4
	258	100BACK	00:54,90	01:06,28	830–471	2–3–4	266	01:00,87	01:18,08	840–395	2–3–4–5
	211	200BACK	02:02,04	02:40,03	771–342	3–4–5	194	02:09,61	02:56,68	861–338	2–3–4–5
	277	50BREAST	00:27,12	00:32,91	876–490	1–2–3–4	274	00:31,10	00:40,04	836–386	2–3–4–5
	279	100BREAST	01:02,22	01:06,28	763–632	3–4	256	01:06,44	01:30,19	899–359	1–2–3–4–5
	235	200BREAST	02:12,73	02:52,19	854–274	2–3–4	183	02:18,41	02:26,82	981–822	1–2
	265	50FLY	00:23,77	00:27,15	822–551	2–3–4	275	00:27,22	00:31,59	722–462	3–4
	249	100FLY	00:53,17	01:01,63	804–516	2–3–4	299	00:59,23	01:13,24	821–434	2–3–4–5
	197	200FLY	01:57,68	02:43,99	824–304	2–3–4	150	02:05,26	03:45,54	919–157	1–2–3–4–5
	223	200IM	02:00,57	02:27,22	845–464	2–3–4	220	02:11,31	02:50,04	886–408	1–2–3–4–5
	171	400IM	04:14,26	05:42,05	882–356	1–2–3–4–5	141	04:32,17	06:25,02	932–329	1–2–3–4–5

AG-1, age-group 1; AG-2, age-group 2; AG-3, age-group 3; AG-4, age-group 4. Level 1: ≥875 FINA points; Level 2: 800–874 FINA points; Level 3: 650–799 FINA points; Level 4: 450–649 FINA points; Level 5: <450 FINA points (18).

The freestyle data were extracted from a previous study (17), which performed similar analyses and procedures. Freestyle data were incorporated exclusively in the comparative analysis between strokes to enable a complete comparison across all individual swimming events. These data were not included in the ANOVA analyses for sex or age group comparisons.

### 2.1 Procedure

Data from all backstroke, breaststroke, butterfly, and individual medley events were extracted from the RFEN ranking. A database was created for each event and organized according to age groups. Swimmers who had a race record only in SC or LC were excluded, meaning that each event included the same swimmers in both SC and LC data, if a swimmer appeared in more than one season, their performances were treated as independent observations for each season. The final sample size for each group is displayed in Table 1.

The differences between SBs in SC and LC for each swimmer were individually calculated by subtracting the SC time from the LC time for each swimmer and the same season (Tdiff).

### 2.2 Statistical analysis

The arithmetic mean ± standard deviation (SD) was obtained for the descriptive analysis of the differences between SB in SC and LC. A *post hoc* power analysis (G*Power 3.1) indicated that with a sample size of 254 participants (mean of swimmers in all the events) and 4 comparison groups and f = 0.45 was approximately 99.99%. The normality of the data distribution for all datasets was checked and confirmed with the Kolmogorov–Smirnov test.

A three-way ANOVA was performed to assess the impact of Event, Sex and Group with a multiple comparison using Bonferroni correction to analyze the differences between each level of factors. The effect size was calculated using partial eta squared (ŋ_p_^2^) and Cohen's d (d). Cohen's effect sizes classified as small (d = 0.20–0.49), medium (d = 0.50–0.79) and large (d ≥ 0.8) (19, 20). Partial eta squared were employed to present the magnitude of main effects or interactions with 0.01, 0.06 an above 0.15 thresholds for the small, medium and large effect, respectively (21).

All analyses were performed using SPSS v. 29.0.1.0 for Mac OS (IBM SPSS Statistics), and PRISM 9 v.9.5 for macOS was used for graphing purposes. The significance level was set at *p* < 0.05.

## 3 Results

The ANOVA revealed that the three-way interactions between event, sex, and group were not significant (*p* = 0.093). However, significant interactions were found in all two-way combinations (*p* = 0.049 for event and sex, *p* < 0.001 for event and group, and *p* = 0.001 for sex and group; Table 2).

**Table 2:** ANOVA results of the differences between SC and LC.

Factor	F	*p*-value	Partial *η*^2^
Event	1,020.127	<0.001	0.314
Sex	0.916	0.338	0
Group	190.455	<0.001	0.025
Event * Sex	1.841	0.049	0.001
Event * Group	11.722	<0.001	0.016
Sex * Group	5.159	0.001	0.001
Event * Sex *Group	1.356	0.093	0.002

### 3.1 Descriptive data about the differences between SC and LC in each swimming event

**Figure 1:**
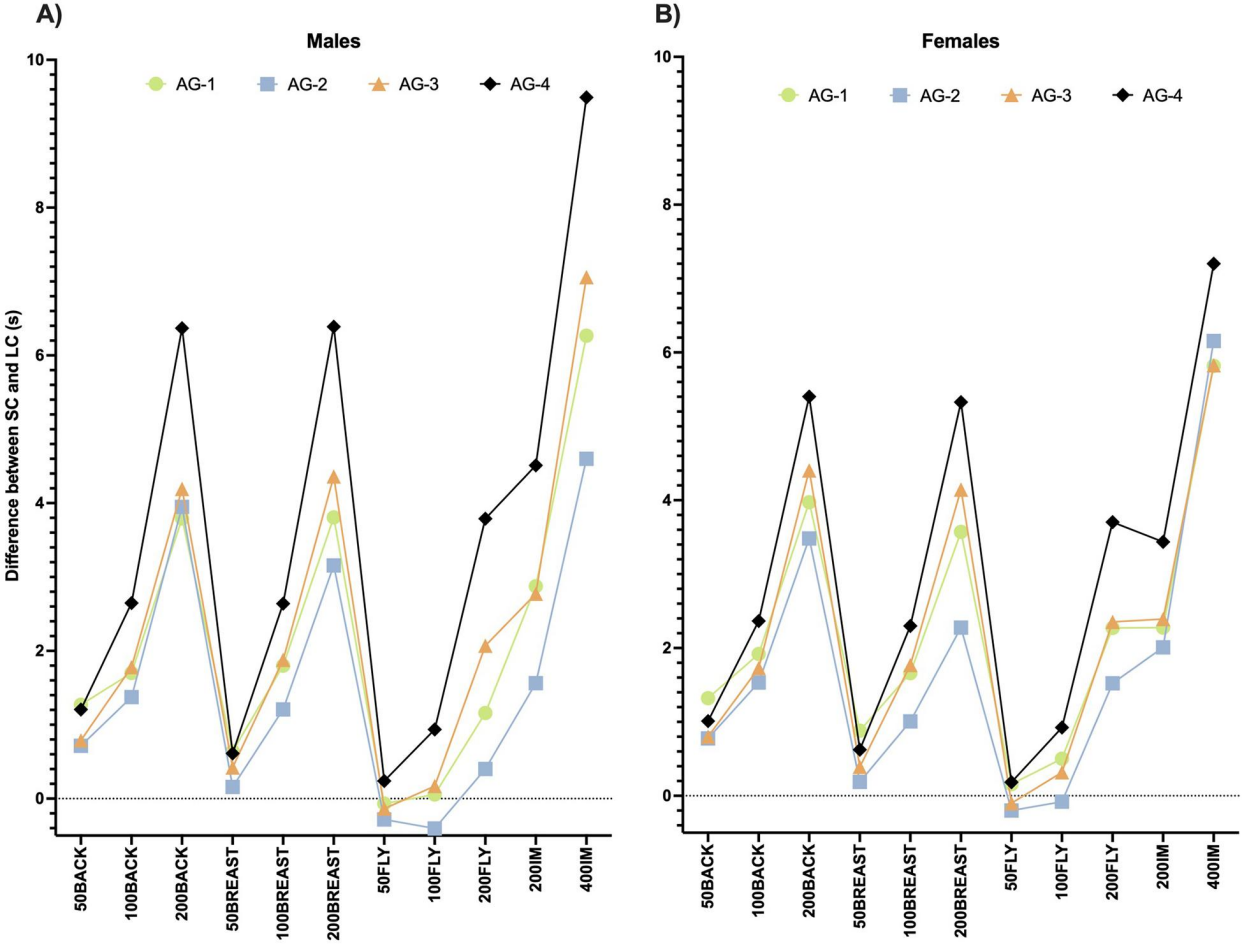
Descriptive data about the differences between SC and LC in each swimming event according to the age groups. **(A)** Male swimmers; **(B)** Female swimmers.

The absolute differences in swimmers’ performance between SC and LC pools exhibited large variations depending on the event (A and B panels). Age group analysis reveals that AG4 tends to exhibit the largest differences in most events, followed by AG3, AG1 and AG2 for both sexes. Interestingly, butterfly events, especially 50 and 100 m, present lower differences in Tdiff than the rest of the strokes in both sexes. However, backstroke and breaststroke events present similar behaviors between them (Figure 1).

### 3.2 Differences in SC and LC times between age-group in each sex

**Figure 2:**
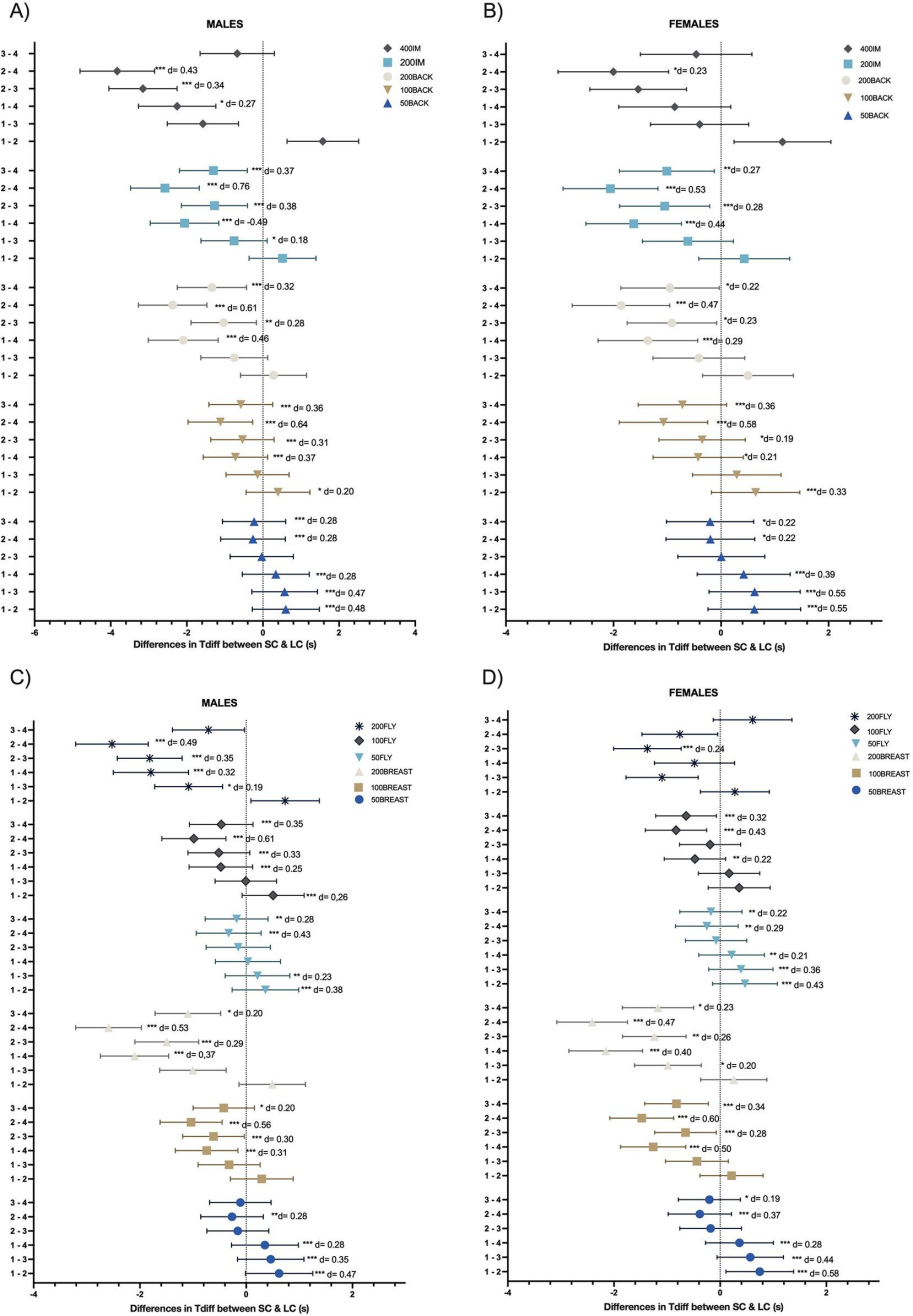
Differences in SC in LC times between age-groups in each sex. Tdiff: Difference in time between the age groups. SC, short Course; LC, long course; **(A)** comparative between male age-groups in backstroke and individual medley events; **(B)** comparative between females age-groups in backstroke and individual medley events; **(C)** comparative between male age-groups in breaststroke and butterfly events; **(D)** comparative between male age-groups in breaststroke and butterfly events; 1,2,3,4: represent age group 1,2,3 and 4 respectively; d = Cohen's D; Significance codes: ***p < 0.001; **p < 0.01; *p < 0.05. Error bars reperesent the 95% condifence interval of the differences

Significant differences in Tdiff were observed among age groups (AG1–AG4) across various swimming events, with AG4 generally showing the highest Tdiff (Figure 2, panels A-D).

In Panel A, AG2, AG3, and AG4 showed significantly higher Tdiff with medium and small effect sizes than AG1 in the 50 m backstroke (*p* < 0.001; d = 0.48, 0.47, and 0.28). In 100 m backstroke, AG2 had significant differences with small and medium effect sizes from AG1 (*p* < 0.05; d = 0.20) and AG4 (*p* < 0.001; d = 0.64), where AG4 showed the highest Tdiff. In the 200 m backstroke, AG2 showed significant differences also with small and medium effect sizes from AG3 (*p* < 0.01; d = 0.28) and AG4 (*p* < 0.001; d = 0.61), again with AG4 presenting the highest Tdiff. Similar results were observed in the individual medley, where AG2 and AG3 showed significant differences with small to medium effect sizes with AG4 in the 200 m and 400 m event (*p* < 0.001; d = 0.38–0.76), with AG4 consistently presenting the greatest Tdiff.

In Panel B, AG2 showed significant differences and small effect size with AG4 in the 400 m individual medley (*p* < 0.05; d = 0.17), linking the larger Tdiff in longer events to AG4. In the 200 m individual medley and 200 m backstroke, AG2 and AG3 showed smaller Tdiff differences and small to medium effect size compared to AG4 (*p* < 0.001; d = 0.28–0.53). AG1 and AG3 also had significant differences with similar effect sizes with AG4 in most events (*p* < 0.001; d = 0.21–0.58). However, in shorter events such as the 50 m backstroke, AG1 and AG2 showed higher Tdiff than AG3 and AG4 (*p* < 0.001; d = 0.22–0.55) with small to medium effect size.

In Panels C and D, AG1 had significantly higher Tdiff with small to medium effect size than AG2 in short-distance events, including the 50 m breaststroke (*p* < 0.001; d = 0.47–0.58) and 50 m butterfly (*p* < 0.001; d = 0.38–0.43). AG3 also presented significant differences and small effect size with AG2 and AG4 in breaststroke and butterfly events (*p* < 0.001; d = 0.20–0.35), confirming AG4 as the group with the highest Tdiff. In longer events, particularly the 100 m breaststroke (*p* < 0.001; d = 0.60), AG4 had the greatest Tdiff with medium effect sizes across all groups. Similarly, AG1 showed significant differences with AG4 in the 200 m breaststroke (males: *p* < 0.001; d = 0.37, females: *p* < 0.05; d = 0.20) and the 200 m butterfly (males: *p* < 0.001; d = 0.32). AG2 also differed significantly with small to medium effect sizes from AG4 in all 200 m events (*p* < 0.001; d = 0.22–0.60) except the butterfly, with AG4 consistently presenting the highest Tdiff across groups.

### 3.3 Differences between backstroke, breaststroke, butterfly and individual medley events between sexes in each age group

**Figure 3:**
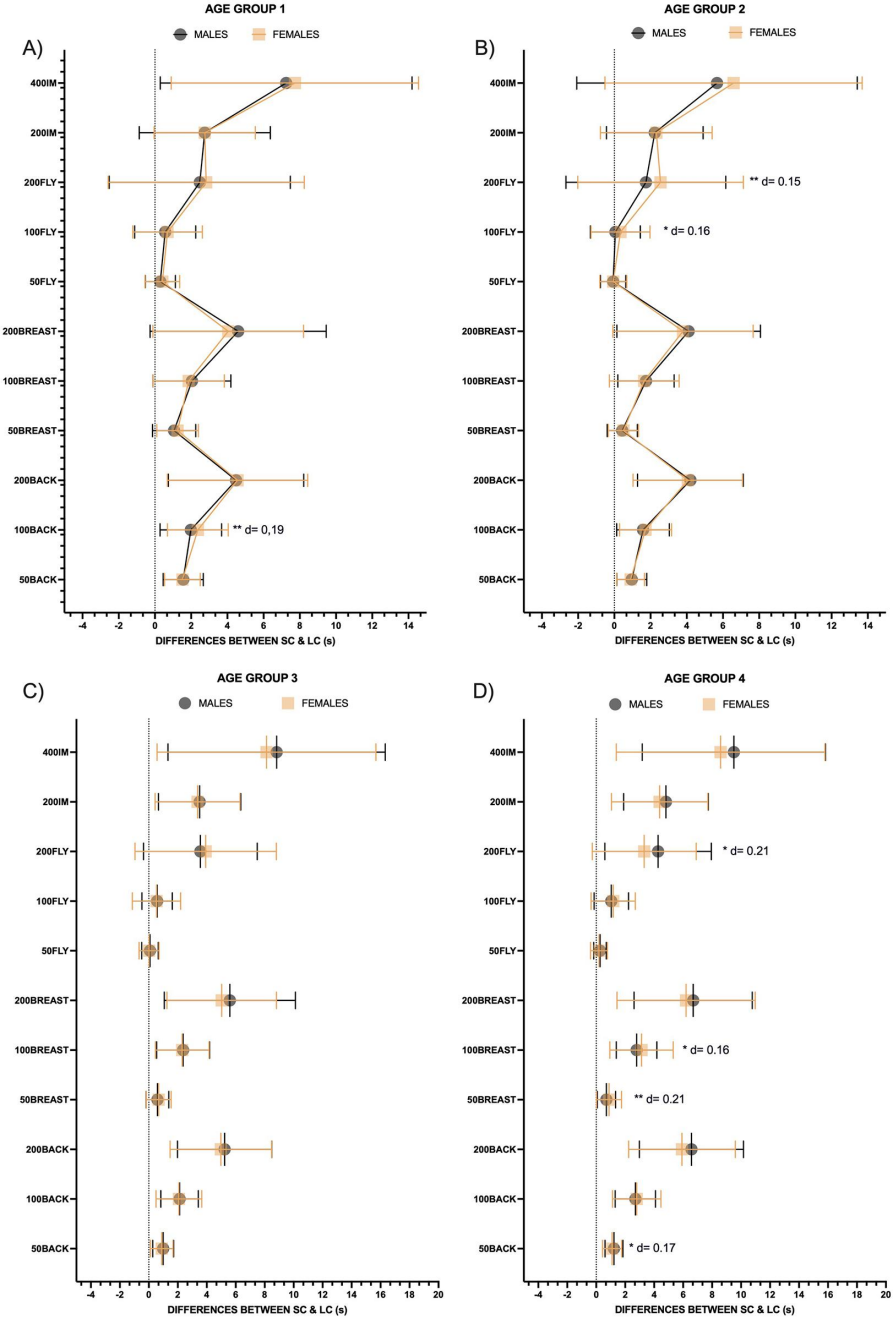
Differences between backstroke, breaststroke, butterfly and Individual medley events between gender in each age-groups. SC, short course; LC, long course; **(A)** Comparative between males and females in age-group 1; **(B)** comparative between males and females in age-group 2; **(C)** comparative between males and females in age-group 3; **(D)** Comparative between males and females in age-group 4; d = Cohen's D; Significance codes: ***p < 0.001; **p < 0.01; *p < 0.05.

Regarding the differences between males and females in each age group (Figure 3), female swimmers had a greater Tdiff than male swimmers in AG1 in the 100 m backstroke (*p* < 0.01; d = 0.19). In AG2, significant differences with small effect sizes (d = 0.15–0.16) were observed in the 200 m butterfly (*p* < 0.01) and 100 m butterfly (*p* < 0.05), with males showing greater Tdiff than females. No significant sex differences were identified in AG3. Finally, AG4 (*p* < 0.05), exhibited the highest Tdiff in the 50 m backstroke (d = 0.17), 100 m breaststroke (d = 0.18) and 200 m butterfly (d = 0.21), as well as in the 50 m breaststroke (*p* < 0.01; d = 0.21). Despite the significance, all the differences were small.

### 3.4 Differences in SC and LC time between the same distance events at each age-group and sex

**Figure 4:**
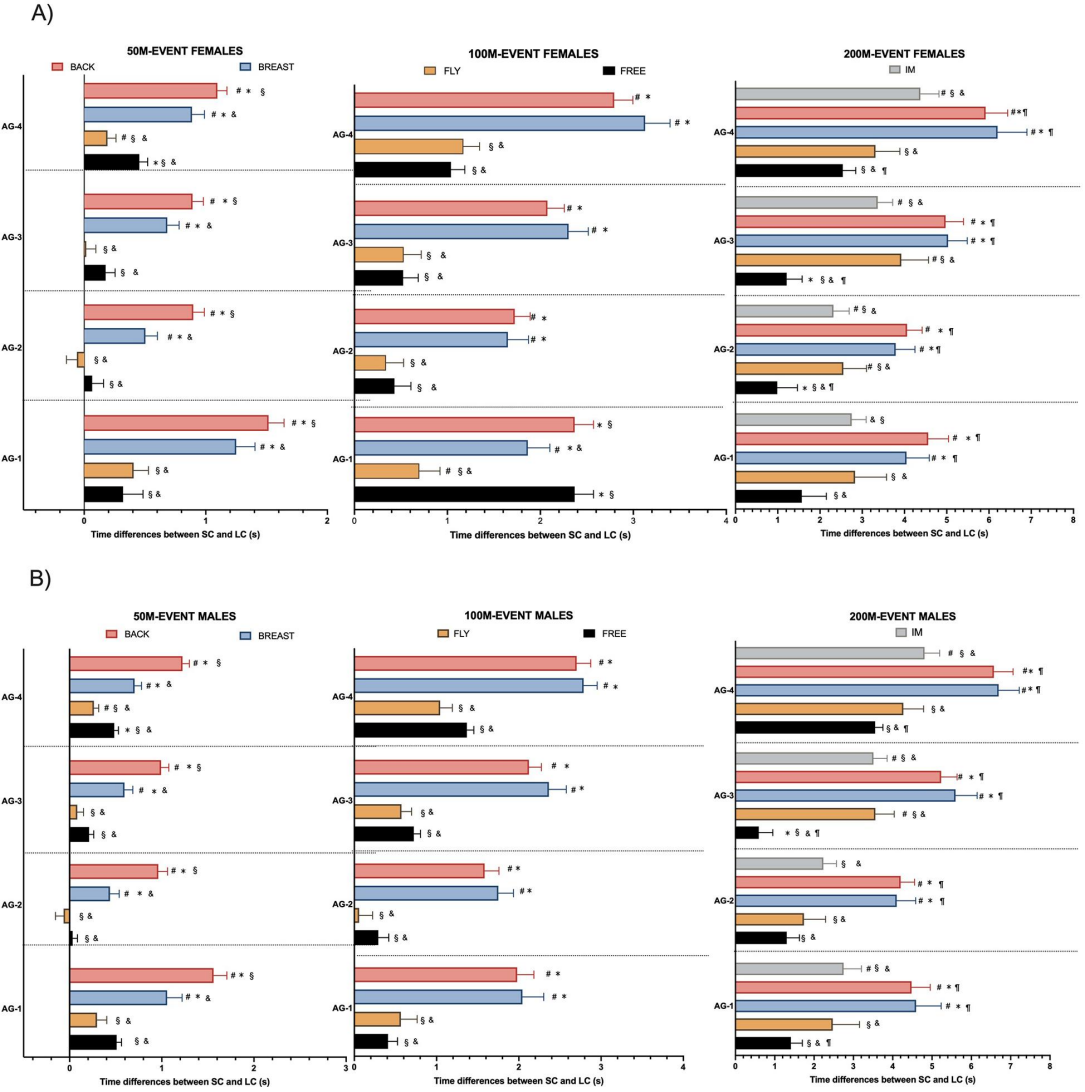
Differences in SC and LC times between same-distance events according to age groups and sex. Figure **(A)** represents male swimmers, and Figure **(B)** represents female swimmers. SC, short course; LC, long course; &: difference with backstroke; *: difference with butterfly; ¶: difference with individual medley; §: difference with breaststroke; #: difference with freestyle. Significance codes: *p* < 0.05.

The differences in same distance events between strokes (Figure 4) showed that, in the 50 m events, butterfly and freestyle exhibited significantly smaller Tdiff compared to backstroke and breaststroke across all age groups and both sexes (*p* < 0.05). In contrast, backstroke showed the largest Tdiff (*p* < 0.05) compared to other swimming strokes.In the 100 m events, backstroke and breaststroke exhibited larger Tdiff compared to butterfly and freestyle across all age groups and sexes (*p* < 0.05), except for AG1 females in which freestyle and backstroke showed similar outcomes. Significant differences between breaststroke and backstroke were found only in AG1 females, where backstroke had a higher Tdiff (*p* < 0.05). Additionally, no differences were observed between freestyle and butterfly, except in AG1 females, where butterfly displayed a smaller Tdiff (*p* < 0.05).

Finally, in the 200 m, the between-stroke differences in Tdiff for both females and males were very similar. Backstroke and breaststroke presented larger Tdiff compared to butterfly and freestyle (*p* < 0.05), and the overall between-stroke difference pattern was consistent across all age groups. In the 200 m individual medley, Tdiff results were similar to those of butterfly, with no significant differences between the two events. When comparing butterfly and freestyle in the 200 m events, Tdiff tended to be greater in butterfly than in freestyle, but significant differences were only found in AG2 and AG3 females and AG3 males (*p* < 0.05).

## 4 Discussion

### 4.1 Differences in SC in LC times between age-group in each sex

The results obtained in the analysis between different ages indicate that senior swimmers (AG4 group) tended to show the greatest Tdiff between SC and LC in most of the events, followed by the AG3, AG1, and AG2 groups for both sexes. Although it was notable that the AG4 group generally presented the largest Tdiff, no clear trends were observed for the other groups. This pattern may be explained by the interplay of multiple factors, as performance tends to evolve over the years due to age-related differences in physiological, morphological, and physical characteristics across age groups (22). Specifically, AG4 showed significant differences from the other groups in most of the backstroke events, 200 m breaststroke, and 400 m Individual Medley. These differences may be attributed to the progressive development of technical and physical capacities with age, such as improved propulsion during turns and greater efficiency in underwater swimming, which enable more experienced swimmers to better exploit the advantages of short course pools (23, 24). Also, from an anthropometric perspective, greater height and longer limbs allow swimmers to cover the same distance with fewer arm stroke cycles, thereby enhancing mechanical efficiency (25). Moreover, these characteristics facilitate the generation of force during each stroke cycle, a benefit that becomes particularly significant in LC events, where free swimming phases are more predominant (26).

Swimmers in AG1 and AG2 do not necessarily exhibit higher stroke rates than those in older age groups, which may make them more reliant on swimming efficiency, as reflected by the stroke index. This greater dependence on efficiency could influence the performance differences observed between SC and LC in these younger age groups (27). The similarities in the mechanics of the stroke suggest that the performance differences between AG1 and AG2 are minimal compared to the older groups, although factors such as cardiorespiratory development may influence the resistance of both groups (28, 29). However, in some cases, AG1 showed a greater Tdiff than AG2. This could be due to AG1's limited experience in long course competitions, which may result in performance differences. Nevertheless, both age groups may face similar challenges in terms of familiarity with the LC format. Additionally, early adolescence is characterized by rapid improvements in performance due to growth and biological maturation. Since SC competitions are typically held at the beginning of the season and LC events later, younger swimmers may perform similarly or even better in LC due to seasonal progression.

When focusing on the older groups (AG3 and AG4), the differences in Tdiff generally increased. This pattern may be linked to physical and technical changes associated with growth and maturation during these stages (30). During puberty, males show more notable increases in height and muscle mass compared to girls, which is associated with more significant improvements in swimming performance. This different developmental pattern between the sexes contributes to progressively widening the gap in athletic performance throughout adolescence and into adulthood. This fact could explain why the differences between AG3 and AG4 are less pronounced than in other combinations (31, 32).

### 4.2 Differences between backstroke, breaststroke, butterfly and individual medley events between sex in each age-group

The comparative analysis between sexes in each swimming event revealed significant differences between male and female swimmers in some age groups and events. Statistically, the differences may be significant. However, their impact on performance is less substantial due to the small effect sizes reported. Thus, these results should be interpreted with caution, as it does not show sufficient consistency to represent a systematic trend. In AG1, female and male swimmers do not have significant differences in Tdiff, practically in all the events in AG1, except for 100 m backstroke, where greater Tdiff differences were observed among females compared to males.

In swimmers from AG2 and AG3, the performance differences in Tdiff were not statistically significant between men and women in many events, suggesting that the impact of the SC vs. LC pool format on performance is comparable between sexes in these age groups, except for AG2 in the 100 and 200 butterfly events, this is probably because the distance underwater could be the reason for longer ripples that allow males to maintain higher speeds between the walls, maximising the benefit of each phase and turn underwater. This results in a greater relative advantage during turns, reducing the overall Tdiff between SC vs. LC (7, 33).

Finally, in senior swimmers AG4, most of the events do not present any differences between male and female swimmers. However, in 200 butterfly males present higher Tdiff compared to females, which may be related to greater strength development in men within this age group, giving them an advantage in SC events where a higher number of undulatory movements, such as in the 200 m, can be a key factor in their superior performance (34). Conversely, in the 50 and 100 m breaststroke events, females displayed higher Tdiff values, a trend not observed in the younger age groups. Nevertheless, all these differences were characterized by small effect sizes and should be interpreted with caution.

### 4.3 Differences in SC and LC time between same distances events in function of ages-groups and sexes

In 50 m events, compared with the other strokes, backstroke had significantly higher differences in the performance achieved in SC and LC, which can be attributed to the biomechanical characteristics, particularly in the underwater phases. However, similar results were not observed in butterfly stroke, even though previous research only shows minor biomechanical differences between dorsal and prone undulatory underwater (35). Thus, the most likely explanation for these differences could be the variations between strokes in the duration and velocities of the underwater phases. For example, previous research indicates that backstroke is the stroke in which swimmers cover the greatest underwater distance during turns (3). In this context, the greater differences between SC and LC swimming could be explained by the fact that, in SC, swimmers spend a larger portion of the race underwater, achieving higher velocities than when swimming on the surface (36, 37). This could contribute to the increased performance differences compared to LC swimming. However, further studies analyzing 50 m events in both LC and SC are needed to confirm that this factor influenced the results.

In the 100 m events, backstroke and breaststroke have significantly greater variations between SC and LC compared to freestyle and butterfly. An interesting outcome was that, unlike the 50 m events, the differences between backstroke and breaststroke were not as clear. Mechanical efficiency could potentially play a key role in this difference between 50 m and 100 m, as breaststroke is the stroke with the lowest efficiency among the four (38). For example, with the low efficiency, the higher proportion of surface swimming in this stroke likely induces faster development of fatigue compared to the other strokes. This lower efficiency may also contribute to the more pronounced differences between SC and LC. In SC, frequent turns offer brief moments of rest for the limbs and provide additional propulsion with each flip, which could perhaps help to mitigate the limitations of the breaststroke. In contrast, in LC, where turns are less frequent, swimmers must rely much more on surface swimming. In these conditions, the mechanical inefficiencies of the breaststroke might become more apparent, contributing to the greater differences observed between SC and LC compared to other strokes.

Extending the analysis to 200 m events, the differences observed in the 100 m between strokes become more pronounced, which might be due to the twice the number of turns or the pacing demands imposed by this distance. According to the literature, frequent turns in SC could help to reduce lactate concentrations, whereas in LC, the pacing becomes more relevant to performance (39) and higher influence of turns on final performance is due to the fact that, as the number of turns increases, the force applied to the wall intensifies and the undulatory effect is enhanced (4). Although the differences in the 200 m events are greater than those in the 100 m, similar patterns were observed across strokes, with comparable differences between SC and LC for backstroke and breaststroke, in contrast to freestyle and butterfly. Regarding individual medley events, previous studies (1) have indicated that in 200 m races for men, backstroke performance is the most influential segment in determining the final result, while in women, backstroke also emerges as the most decisive stroke within the overall event. These findings highlight the critical role of backstroke in longer distance races, particularly in relation to the cumulative impact of turns and the technical demands associated with underwater phases.

## 5 Conclusion

In summary, this study demonstrated that performance differences between SC and LC vary depending on stroke, event, age group, and sex. The largest SC–LC differences were observed in backstroke and breaststroke events, particularly at 200 m distances, and were more pronounced in older swimmers (AG4). In contrast, smaller differences were found in freestyle and butterfly events across all age groups. These findings highlight that performance variation between SC and LC is influenced by both stroke type and age, indicating that comparative analyses across pool lengths should be adjusted accordingly.

## 6 Limitations

The principal limitation of this study is that it cannot be controlled whether the best performances in SC and LC occurred within the same competitive period, which may introduce variability due to seasonal or training-related factors. Additionally, the freestyle data were extracted from a previous study that included only the top 100 Spanish swimmers, potentially reducing the sample size in some categories and slightly limiting the statistical power for those events. Both factors should be considered when interpreting the results.

## Data Availability

The raw data supporting the conclusions of this article will be made available by the authors, without undue reservation.

## References

[1] González-Ravé JM Santos-Cerro J González-Megía P Pyne D. Contributions of each of the four swimming strokes to elite 200–400 individual medley swimming performance in short and long course competitions. PeerJ. (2023) 11:e16612. 10.7717/peerj.16612 38111656 PMC10726738

[2] Craig A Boomer W Skehan P. Patterns of velocity in competitive breaststroke swimming. Swimming Science V. (1988) 18:73–7.

[3] Veiga S Roig A Gómez-Ruano MA. Do faster swimmers spend longer underwater than slower swimmers at world championships? Eur J Sport Sci. (2016) 16(8):919–26. 10.1080/17461391.2016.1153727 26930126

[4] Marinho DA Barbosa TM Neiva HP Silva AJ Morais JE. Comparison of the start, turn and finish performance of elite swimmers in 100m and 200m races. J Sports Sci Med. (2020) 19(2):397–407. 32390734 PMC7196746

[5] Weimar W Sumner A Romer B Fox J Rehm J Decoux B Kinetic analysis of swimming flip-turn push-off techniques. Sports. (2019) 7(2):32. 10.3390/sports7020032 30696072 PMC6409673

[6] Born D-P Romann M Stöggl T. Start fast, swim faster, turn fastest: section analyses and normative data for individual medley. J Sports Sci Med. (2022) 21(2):233. 10.52082/jssm.2022.233 35719225 PMC9157519

[7] Veiga S Cala A Frutos PG Navarro E. Comparison of starts and turns of national and regional level swimmers by individualized-distance measurements. Sports Biomech. (2014) 13(3):285–95. 10.1080/14763141.2014.910265 25325772

[8] Polach M Thiel D Kreník J Born D-P. Swimming turn performance: the distinguishing factor in 1500m world championship freestyle races? BMC Res Notes. (2021) 14(1):248. 10.1186/s13104-021-05665-x 34193247 PMC8243611

[9] Wolfrum M Knechtle B Rüst CA Rosemann T Lepers R. The effects of course length on freestyle swimming speed in elite female and male swimmers—a comparison of swimmers at national and international level. SpringerPlus. (2013) 2(1):643. 10.1186/2193-1801-2-643 24349949 PMC3862862

[10] Keskinen OP Keskinen KL Mero AA. Effect of pool length on blood lactate, heart rate, and velocity in swimming. Int J Sports Med. (2007) 28(05):407–13. 10.1055/s-2006-924505 17111309

[11] Lowensteyn I Perry A Nash M Salhanich D. Differences in peak blood lactate concentration in long course versus short course swimming. J Swim Res. (1994) 10:31–4. 10.5604/17342260.1068225

[12] Wirtz W Wilke K Zimmermann F. Velocity, distance per stroke and stroke frequency op highly skilled swimmers in 50 m freestyle sprint in a 50 and 25 m pool. In: Biomechanics and Medicine in Swimming V1. London: Taylor & Francis (2013). p. 113–6.

[13] Schaub P Rüst CA Rosemann T Knechtle B. Differences in swimming speed between short and long course from 50m to 1,500m in elite female and male Swiss freestyle age group swimmers. Adapt Med. (2015) 7(4):186–95. 10.4247/AM.2015.ABF125

[14] Ratel S Blazevich AJ. Are prepubertal children metabolically comparable to well-trained adult endurance athletes? Sports Med. (2017) 47(8):1477–85. 10.1007/s40279-016-0671-1 28044282

[15] Demirkan E Özkadı T Alagöz İ Çağlar EÇ Çamiçi F. Age-related physical and performance changes in young swimmers: the comparison of predictive models in 50-meter swimming performance. Balt J Health Phys Act. (2023) 15(2):4. 10.29359/BJHPA.15.2.04

[16] Morais JE Barbosa TM Forte P Silva AJ Marinho DA. Young swimmers’ anthropometrics, biomechanics, energetics, and efficiency as underlying performance factors: a systematic narrative review. Front Physiol. (2021) 12:691919. 10.3389/fphys.2021.691919 34603070 PMC8481572

[17] Hermosilla F Beltrán-Díaz E Gonjo T Juárez Santos-García D. Differences in seasonal best times between short and long course in freestyle events in Spanish age-group swimmers. J Sports Sci. (2023) 41(6):557–64. 10.1080/02640414.2023.2227540 37356108

[18] Ruiz-Navarro JJ López-Belmonte Ó Gay A Cuenca-Fernández F Arellano R. A new model of performance classification to standardize the research results in swimming. Eur J Sport Sci. (2023) 23(4):478–88. 10.1080/17461391.2022.2046174 35193458

[19] Cohen J. Statistical Power Analysis for the Behavioral Sciences. 2nd ed. New York, NY: Routledge (2013). Available online at: 10.4324/9780203771587

[20] Cohen J. Statistical power analysis. Curr Dir Psychol Sci. (1992) 1(3):98–101. 10.1111/1467-8721.ep10768783

[21] Cohen J. Statistical Power Analysis for the Behavioral sciences. New York, NY: Routledge (1988).

[22] Morais JE Silva AJ Marinho DA Lopes VP Barbosa TM. Determinant factors of long-term performance development in young swimmers. Int J Sports Physiol Perform. (2017) 12(2):198–205. 10.1123/ijspp.2015-0420 27196954

[23] Hermosilla F Sanders R González-Mohíno F Yustres I González-Rave JM. Effects of dry-land training programs on swimming turn performance: a systematic review. Int J Environ Res Public Health. (2021) 18(17):9340. 10.3390/ijerph18179340 34501929 PMC8431432

[24] Jones JV Pyne DB Haff GG Newton RU. Comparison between elite and subelite swimmers on dry land and tumble turn leg extensor force-time characteristics. J Strength Cond Res. (2018) 32(6):1762–9. 10.1519/JSC.0000000000002041 29786631

[25] González-Ravé JM González-Mohino F Hermosilla Perona F Rodrigo-Carranza V Yustres I Biomechanical PD. Physiological and anthropometric determinants of backstroke swimming performance: a systematic review. Sports Med Open. (2025) 11(1):68. 10.1186/s40798-025-00868-z 40483624 PMC12146239

[26] Alves M Carvalho DD Fernandes RJ Vilas-Boas JP. How anthropometrics of young and adolescent swimmers influence stroking parameters and performance? A systematic review. Int J Environ Res Public Health. (2022) 19(5):2543. 10.3390/ijerph19052543 35270236 PMC8909379

[27] Morais JE Jesus S Lopes V Garrido N Silva A Marinho D Linking selected kinematic, anthropometric and hydrodynamic variables to young swimmer performance. Pediatr Exerc Sci. (2012) 24(4):649–64. 10.1123/pes.24.4.649 23196769

[28] Nikitakis IS Paradisis GP Bogdanis GC Toubekis AG. Physiological responses of continuous and intermittent swimming at critical speed and maximum lactate steady state in children and adolescent swimmers. Sports (Basel). (2019) 7(1):25. 10.3390/sports7010025 30669295 PMC6359490

[29] Naughton G Farpour-Lambert NJ Carlson J Bradney M Van Praagh E. Physiological issues surrounding the performance of adolescent athletes. Sports Med. (2000) 30(5):309–25. 10.2165/00007256-200030050-00001 11103846

[30] Vorontsov A. Swimming speed, stroke rate and stroke length during maximal 100 m freestyle of boys 11–16 years of age. In: Chatard JC, editor. Biomechanics and Medicine in Swimming IX. Saint-Étienne: Publications de l'Université de Saint-Étienne (2003). p. 195–9.

[31] Peulić J Obradović A Jurišić MV Obradović J. The influence of anthropometric characteristics on swimming speed in adolescent swimmers. Exercise and Quality of Life. (2023) 15(2):33–40. 10.31382/eqol.231204

[32] Ferraz R Branquinho L Loupo R Neiva H Marinho DA. The relationship between anthropometric characteristics and sports performance in national-level young swimmers. Eur j hum mov. (2020) 45:45. 10.21134/eurjhm.2020.45.2

[33] Seifert L Boulesteix L Chollet D Vilas-Boas J. Differences in spatial-temporal parameters and arm–leg coordination in butterfly stroke as a function of race pace, skill and gender. Hum Mov Sci. (2008) 27(1):96–111. 10.1016/j.humov.2007.08.001 17935810

[34] Born D-P Kuger J Polach M Romann M. Start and turn performances of elite male swimmers: benchmarks and underlying mechanisms. Sports Biomech. (2024) 23(4):484–502. 10.1080/14763141.2021.1872693 33663342

[35] Arellano R Garcia F Gavilan A. Comparison of the underwater undulatory swimming technique in two different body positions. In: Keskinen KL Komi PV Hollander AP, editors. Biomechanics and Medicine in Swimming VIII. Jyväskylä (Finland): Gummerus Printing (1999). p. 25–8.

[36] Veiga S Qiu X Trinidad A Dolek BE De la Rubia A Navarro E. Effect of the skill, gender, and kick order on the kinematic characteristics of underwater undulatory swimming in the dorsal position. J Hum Kinet. (2024) 90:45–56. 10.5114/jhk/168600 38380311 PMC10875688

[37] De Jesus K De Jesus K Gonçalves P Vasconcelos MO Medeiros AIA Carvalho DAD Lateral kinetic proficiency and asymmetry in backstroke start performed with horizontal and vertical handgrips. Sports Biomech. (2021) 20(1):71–85. 10.1080/14763141.2018.1522368 30422057

[38] Kolmogorov S Vorontsov A Vilas-Boas JP. Metabolic power, active drag, mechanical and propelling efficiency of elite swimmers at 100 meter events in different competitive swimming techniques. Appl Sci. (2021) 11(18):8511. 10.3390/app11188511

[39] Janoschka A. The effect of 25 M versus 50 M course length on backstroke performance—an analysis of national and international swimmers. J Athl Enhanc. (2014) 3(1):1. 10.4172/2324-9080.1000137

